# Analysis of factors associated with axial length development in patients with unilateral congenital cataract

**DOI:** 10.1186/s12886-026-04614-1

**Published:** 2026-01-24

**Authors:** Jianling Yang, Xin Zhong, Yi Shi, Lichun Wu, Yuqing Wu, Geng Wang

**Affiliations:** Department of Cataract, Joint Shantou International Eye Center of Shantou University and The Chinese University of Hong Kong, Shantou, 515041 China

**Keywords:** Axial length, Congenital cataract, Ocular development, Form-deprivation

## Abstract

**Purpose:**

To investigate the factors associated with axial length (AL) development in patients with unilateral congenital cataract.

**Methods:**

This retrospective study reviewed clinical records of children with unilateral congenital cataract who underwent surgery at the Joint Shantou International Eye Center from October 2014 to October 2019. Multiple linear regression analysis was utilized to assess the impact of different factors on axial length differences (ALD). Statistical analysis was performed using SPSS software version 29.

**Results:**

A total of 81 patients were included, aged 5.5 months to 14 years (mean 4.86 ± 3.54 years), including 45 females and 36 males. The AL was significantly longer in affected eyes across all groups (*p* = 0.003; *p* < 0.001; *p* = 0.015; *p* < 0.001, respectively). A positive correlation between ALD and duration of form deprivation was observed in the 0-1year group (*r* = 0.679, *p* = 0.031)), whereas a negative correlation was found in the combined age groups of 1–14 years (*r*=-0.322, *p* = 0.006). Multiple linear regression analysis revealed that duration of form deprivation (β =-0.307, *p* = 0.015, 95%CI: [-0.187, -0.021]), degree of lens opacity (β = 0.374, *p* = 0.041, 95%CI: [0.042, 1.855]), and history of oxygen exposure (β = -0.315, *p* = 0.025, 95%CI: [-1.843, -0.125]) were significantly associated with ALD.

**Conclusion:**

The AL was significantly longer in affected eyes across all groups. The duration of form deprivation, degree of lens opacity, and history of oxygen exposure collectively contribute to ALD. Early detection and intervention are crucial to prevent abnormal ocular development in children with congenital cataract.

## Introduction

Axial length (AL) plays a crucial role in assessing eyeball development, influenced by various factors [1, 2]. Congenital cataract, a leading cause of pediatric blindness, impacts the development of the globe, with pathological changes in AL attributed to defocusing and visual form deprivation [3, 4]. Animal experiments have revealed that early visual form deprivation can lead to increased axial length by disrupting the normal feedback mechanisms that regulate ocular growth. This disruption may result in scleral remodeling and an increase in vitreous chamber depth. Additionally, reduced retinal light stimulation alters growth signals transmitted from the retina to the brain, further promoting axial elongation [5, 6]. Clinical studies have reported inconsistent findings regarding axial length (AL) in patients with monocular congenital cataract. While some studies observed that the AL of the affected eye is shorter than that of the fellow eye [7, 8], others reported that the affected eyes can have either longer or shorter AL compared to the normal fellow eye. These discrepancies may be attributed to variations in patient age, duration of form deprivation, severity of lens opacity, and differences in study design or measurement techniques [9]. Moreover, individual compensatory mechanisms and external environmental factors could also play a role in these variations [3, 10, 11]. It’s reported that axial length development is affected by form deprivation resulting from cataracts; [12–14] however, there is a notable lack of published information specifically describing the degree of lens opacity and its relationship with eyeball development. Furthermore, reports on the factors influencing ALD in unilateral congenital cataracts are rare. Besides, the ALD between the affected eye and the normal fellow eye is essential not only for assessing eyeball development but also a significant reference in calculating intraocular lens power and predicting treatment prognosis [15, 16]. In this retrospective study, we aimed to investigate the various factors associated with the ALD in patients with unilateral congenital cataract.

## Methods

### Study design and participants

We conducted a retrospective review of the medical records of patients who diagnosed with unilateral congenital cataract and underwent surgical intervention at the Joint Shantou International Eye Center from October 2014 to October 2019. This study adhered to the STROBE guidelines for reporting observational studies. The diagnosis of unilateral congenital cataract was made based on a combination of clinical features, including lens opacity observed under slit-lamp biomicroscopy and a history of impaired visual function since infancy. For children who could cooperate, a slit-lamp examination was performed. For younger patients, particularly those less than 2 years old who were unable to cooperate, a speculum was used to gently hold the eyelids open, and the diagnosis was confirmed under a microscope. Inclusion criteria were complete medical records including AL measurements for both the affected and fellow eyes. Other ocular diseases, including traumatic cataracts, cataracts secondary to genetic disorders, or other systemic syndromes, were excluded from this study. Patients with bilateral congenital cataract were also excluded, resulting in a final sample of 81 patients. A post-hoc power analysis indicated that the sample size of 81 patients provided sufficient power (> 80%) to detect significant associations in the regression analysis. This study is a retrospective analysis based on anonymized medical records. Since no identifiable private information was used, the requirement for informed consent was waived. The study adhered to the principles of the Declaration of Helsinki and was approved by the Institutional Review Board at the Joint Shantou International Eye Center, Shantou, China (EC 201906123-P20).

### Data collection

The data collected included demographic information, duration of form deprivation (age at surgery), degree of lens opacity, and history of oxygen exposure at birth. Axial length measurements were obtained using contact A-scan ultrasonography.

Patients were divided into four age groups for analysis: 0–1 year, 1–3 years, 3–5 years, and 5–14 years. The age grouping was based on the age at surgery. This grouping was determined based on developmental stages and clinical considerations in pediatric patients.

Specifically:

1) 0–1 year: Refers to infants from birth up to and including 1 year (0 ≤ age ≤ 1 year), representing the critical early period of ocular development during infancy.

2) 1–3 years: Refers to children older than 1 year but not exceeding 3 years (1 < age ≤ 3 years), covering toddlers undergoing rapid sensory and motor development.

3) 3–5 years: Refers to children older than 3 years but not exceeding 5 years (3 < age ≤ 5 years), including preschool-aged children, marking a transitional phase in visual and physical growth.

4) 5–14 years: Refers to children older than 5 years but not exceeding 14 years (5 < age ≤ 14 years), encompassing school-aged children during a period when ocular growth typically stabilizes.

#### A-scan examination

AL measurements from both the affected and normal fellow eyes were obtained using a contact A-scan (B-SCAN-Vplus/BIOVISION, Quantel Medical, France). The A-scan unit featured a 10 MHz transducer probe, with velocities set to 1,641 m/s for the cornea and lens and 1,532 m/s for the aqueous and vitreous. All examinations were performed by the same experienced examiner. For patients unable to cooperate, sedation with 10% chloral hydrate (0.8 ml/kg, administered orally or rectally) was employed following pediatric consultation. After instilling one drop of topical anesthetic (0.5% Alcaine, Alcon, USA) into the conjunctival sac, ultrasound applanation was conducted. Each eye was measured 10 times, and the average value was obtained.

#### Degree of lens opacity

Given the inherent subjectivity in assessing the degree of cataract opacity in medical records, we reviewed all the available surgical videos (72 out of 81) to double-confirm the lens opacity in each cataractous eye. Based on the degree of lens opacity, the 72 eyes were classified into two groups: complete cataract group (28 eyes) and partial cataract group (44 eyes). Complete cataract was defined as dense opacity that obscures the entire area of the pupil when fully dilated during surgery. Partial cataract was defined as non-axial (partially obscuring the pupil) or non-dense opacity.

### Statistical analysis

The statistical analysis was carried out using SPSS software version 29 (IBM Corp, Armonk, NY, USA). Qualitative variables were described using absolute frequencies (n) and relative frequencies (%), while mean and standard deviation (mean ± SD) were utilized for the description of age (duration of form deprivation), AL and ALD. The normality of the distribution for all variables was assessed using the Kolmogorov-Smirnov test. A post-hoc power analysis indicated that the sample size of 81 patients provided sufficient power (> 80%) to detect significant associations in the regression analysis.

Pair t-tests were used to compare AL between the affected and fellow eyes. ALD was calculated as the difference in AL between the affected and fellow eyes. Pearson correlation coefficients were computed to assess the relationship between ALD and various factors, including the duration of form deprivation, degree of lens opacity, and history of oxygen exposure. Variables showing potential association (*p* < 0.1) or clinical significance were entered into the multiple linear regression model. Multiple regression analysis was employed to identify the combined effects of these factors on ALD. A P value<0.05 was considered statistically significant.

## Results

### Demographic and clinical characteristics

The study cohort consisted of 81 children with unilateral congenital cataract, aged 5.5 months to 14 years (mean age 4.86 ± 3.54 years), with a gender distribution of 45 females and 36 males. All variables follow a normal distribution. Axial length measurements revealed that the affected eyes had a significantly longer AL compared to the fellow eyes across all age groups, as demonstrated by paired t-test results (t-statistic values: t = 0.793, *p* = 0.003; t = 0.875, *p* = 0.001; t = 0.554, *p* = 0.015; t = 0.571, *p* < 0.001, respectively) (Table 1). Although the axial length (AL) of the affected eyes was significantly longer on average, 21 patients (25.9%) exhibited shorter AL in the cataract eye compared to the contralateral eye. These cases were distributed across different age groups: 1 patient (10%) in Group 1 (0–1 year), 6 patients (31.6%) in Group 3 (3–5 years), and 14 patients (43.8%) in Group 4 (5–14 years). The AL differences in these cases ranged from − 0.11 mm to -2.15 mm.

**Table 1:** Comparison of the AL between affected and fellow eyes in four age groups

	0–1 Years	1–3 Years	3–5 Years	5–14 Years
AL of Affected eye (mm)	19.98 ± 2.05	21.92 ± 1.53	22.78 ± 1.71	23.38 ± 1.65
AL of Fellow eye (mm)	19.56 ± 1.11	21.03 ± 1.38	22.24 ± 0.84	23.00 ± 1.21
ALD	0.42 ± 1.50	0.90 ± 0.49	0.53 ± 1.44	0.39 ± 1.38
n	10	20	19	32
t	0.793	0.875	0.554	0.571
p	<0.01	<0.01	<0.05	<0.01

The data are presented as the means ± standard deviation (SD). Bold data are significant at *p*<0.05; AL: axial length; ALD: axial length differences; n: number; t: t-statistic from paired t-tests comparing AL between affected and fellow eyes in each age group

### Distribution of complete and partial cataracts

As the age of pediatric patients increased, the proportion of complete cataracts tended to decrease in the corresponding age groups, while the proportion of partial cataracts showed an increasing trend, as demonstrated in Table 2.

**Table 2:** Distribution of complete and partial cataract in four age groups

	0–1 Years	1–3 Years	3–5 Years	5–14 Years
**Lens opacity**				
n	9	20	15	28
Complete	9/9	12/20	1/15	6/28
Partial	0/9	8/20	14/15	22/28

n: number

### Correlation analysis

**Fig. 1:**
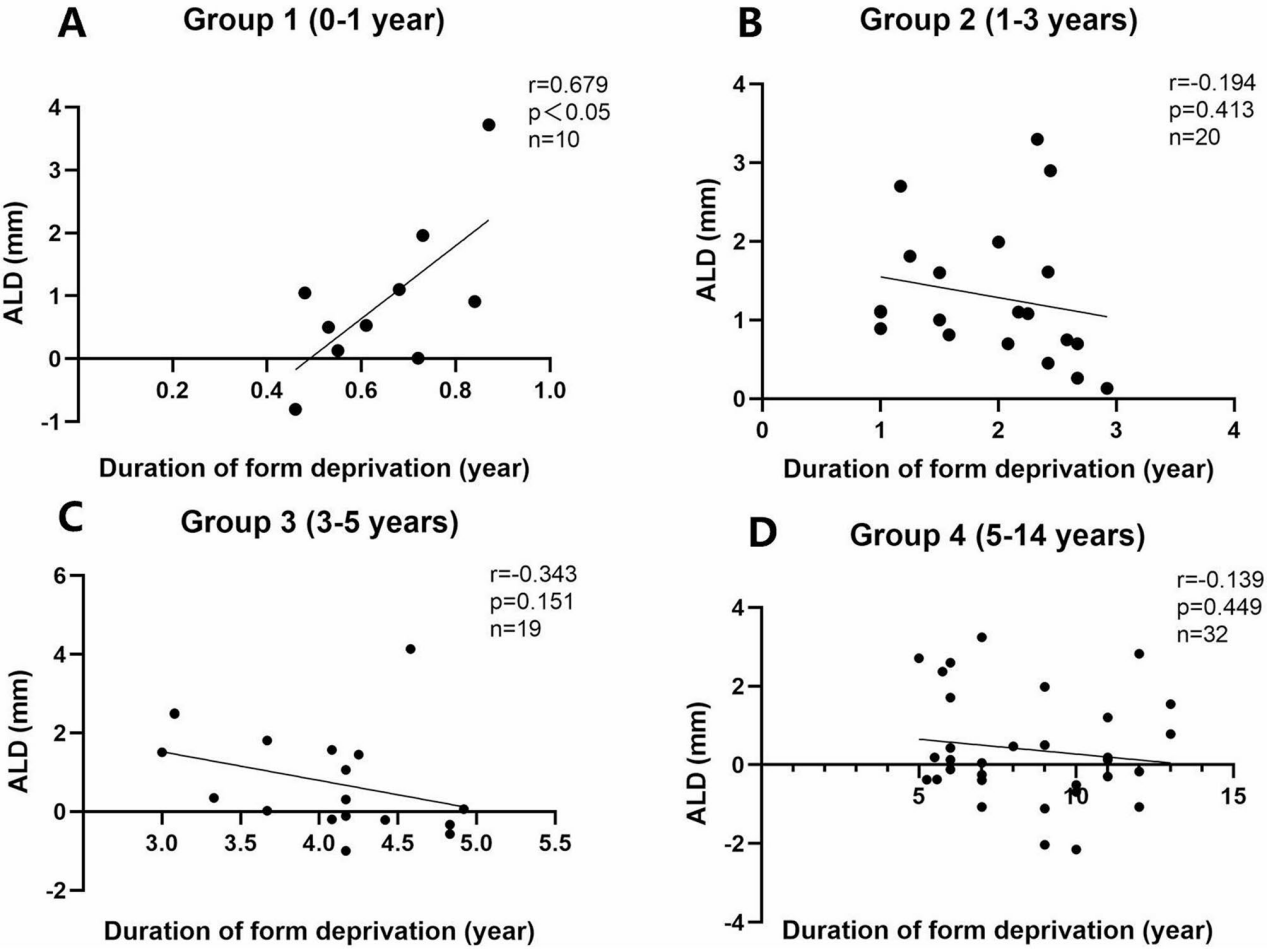
**A-D**. Correlation between ALD and duration of form deprivation in different age groups. (**A**) Group 1 (0–1 year): A positive correlation was observed between ALD and duration of form deprivation (*r* = 0.679, *p* < 0.05, *n* = 10). (**B**) Group 2 (1–3 years): No statistically significant correlation was detected (*r*=-0.194, *p* = 0.413, *n* = 20). (**C**) Group 3 (3–5 years): No statistically significant correlation was detected (*r*=-0.343, *p* = 0.151, *n* = 19). (**D**) Group 4 (5–14 years): No statistically significant correlation was detected (*r*=-0.139, *p* = 0.449, *n* = 32). ALD: axial length differences

**Fig. 2:**
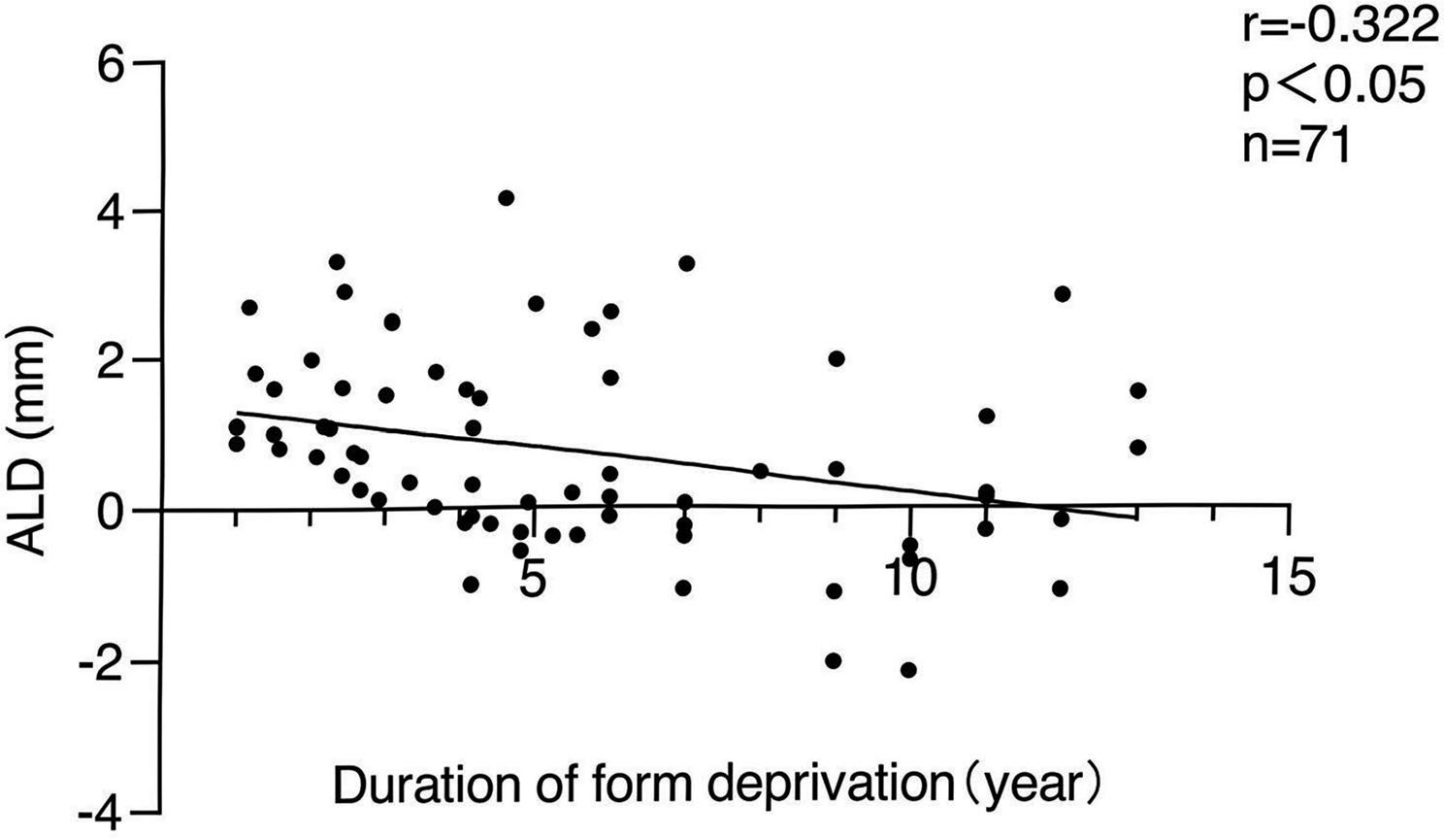
Correlation between duration of form deprivation and ALD in group 2–4 (aged 1–14 years). *p* < 0.05 was statistically significant. ALD: axial length differences

In the 0-1year age group, a significant positive correlation was observed between ALD and the duration of form deprivation (*r* = 0.679, *p* = 0.031) (Fig. 1A). In the 1–3 years age group, no statistically significant correlation was found (*r*=-0.194, *p* = 0.413, *n* = 20) (Fig. 1B). Similarly, in the 3–5 years age group, the correlation was negative but not statistically significant (*r*=-0.343, *p* = 0.151, *n* = 19) (Fig. 1C). In the 5–14 years age group, a weak negative correlation was observed but was not statistically significant (*r*=-0.139, *p* = 0.449, *n* = 32) (Fig. 1D). Conversely, when the data from the 1–14 years age group were combined, a statistically significant negative correlation was detected (*r*=-0.322, *p* = 0.006, *n* = 71) (Fig. 2). Multiple linear regression analysis revealed that the duration of form deprivation, degree of lens opacity, and history of oxygen exposure at birth significantly were associated with the ALD between the affected and fellow eyes (R²=0.228; Duration of form deprivation: standardized beta=-0.307, *p* = 0.015, 95%CI: [-0.187,0.021]; Degree of lens opacity: standardized beta = 0.374, *p* = 0.041, 95%CI: [0.042, 1.855]; History of oxygen exposure: standardized beta=-0.315, *p* = 0.025, 95%CI: [-1.843, -0.125], respectively). In the 0-1year age group, a significant positive correlation was observed between ALD and the duration of form deprivation (*r* = 0.679, *p* = 0.031) (Fig. 1A).

## Discussion

This study involved a comparison of the axial length (AL) between the affected and fellow eyes in patients aged 0–14 years diagnosed with unilateral congenital cataract. The AL of the affected eyes was longer than that of the fellow eyes across all groups. This outcome is consistent with some previous studies [10, 17, 18], but differs from others [7, 8, 19]. These differences may stem from variations in study design, grouping strategies, measurement techniques, or participants characteristics, such as age distribution and the proportion of complete and partial cataracts.

Interestingly, in this study, 21 patients (25.9%) exhibited shorter AL in the cataract-affected eye compared to the contralateral eye. Most of these cases were observed in Group 3 (3–5 years) and 4 (5–14 years), suggesting that older children may exhibit greater variability in AL development. This could be attributed to prolonged form deprivation, delayed treatment, or compensatory mechanisms, such as scleral remodeling. These findings highlight the heterogeneity of AL development in unilateral congenital cataract and underscore the need for further investigation with larger and more diverse cohorts.

The significant positive correlation observed in the 0–1-year age group underscores the critical influence of early form deprivation on axial elongation. During this key development period, the lack of visual input disrupts normal ocular growth feedback mechanisms, leading to excessive AL growth. In contrast, no statistically significant correlations were observed in the 1–3 years, 3–5 years, and 5–14 years age groups, suggesting that as the eye matures, the effects of form deprivation may become less pronounced or are modulated by other compensatory mechanisms. However, when the data for the 1–14 years age group were combined, a significant negative correlation emerged between ALD and the duration of form deprivation. This suggests that the cumulative effects of prolonged deprivation in older children may differ from those in younger children, potentially reflecting compensatory mechanisms or differing growth trajectories.

Our findings are consistent with earlier research indicating that AL in unilateral cataract patients changes rapidly during the first 18 months of life and then plateaus in older children [20]. This can be partly explained by previous research showing that visually normal infants’ eyes perceive only low spatial frequencies [21, 22], while children deprived of vision during the first 6 months fail to develop normal sensitivity to high-spatial frequencies later in life [23]. These results emphasize the importance of early detection and timely intervention to mitigate adverse effects on ocular development.

To the best of our knowledge, this is the first study to utilize cataract surgery videos for confirming the degree of lens opacity. We observed that the proportion of complete cataracts tended to decrease with age, while partial cataracts increased. An explanation might be that complete cataract opacity, causing leukocoria, are more readily observed by parents, prompting earlier medical consultation.

The association between increased AL and prolonged form deprivation is generally recognized [3, 6]. Our results demonstrate that the duration of form deprivation, the degree of lens opacity, and the history of oxygen exposure at birth have a significant impact on the axial length difference (ALD) between the affected and fellow eyes. Possible mechanisms include the crucial interactions between the eyes and light perception during early development, which are essential for the formation of eye-specific domains within the lateral geniculate nucleus and the visual cortex [24, 25]. A cloudy lens can disrupt this light reception or prevent light from focusing on the retina, thereby impairing the transmission of visual signals to the visual centers and ultimately affecting ocular development [26, 27]. Additionally, premature infants, who have severely reduced antioxidant defenses, are particularly sensitive to the toxic effects of oxygen. Oxygen-induced metabolic changes within the retina may accelerate photoreceptor cell loss [28]. Oxygen also triggers abnormal physiological responses, such as retinal vasoconstriction, disrupting light signal transmission and resulting in irregular ocular development [28, 29]. 

This study has several limitations. Firstly, the axial length measurements in this study were obtained using contact A-scan ultrasonography, which is susceptible to variability depending on the technician’s skill and represents a more subjective method compared to the automated and non-contact method like IOL Master. Also, previous studies have suggested that contact A-scan measurements may result in shorter AL values compared to immersion A-scan in pediatric eyes due to corneal compression [30]. For instance, Tadros et al. [8]. reported AL values of 22.2 ± 2.0 mm in affected eyes and 23.0 ± 1.3 mm in fellow eyes in monocular congenital cataract patients aged 8.4 ± 2.8 years, while Borghol-Kassar et al. [31]. found AL values of 22.81 ± 2.74 mm in affected eyes and 23.09 ± 0.95 mm in fellow eyes in patients aged 12.5 ± 5.8 years. Nevertheless, this discrepancy was mitigated by using the same apparatus consistently across all subjects. Secondly, we enrolled patients with comprehensive medical records, including the axial length measurements for both the affected eye and the fellow eye. As a result, those with incomplete clinical data were excluded, potentially introducing a minor yet unavoidable selection bias. Thirdly, in our review of clinical data, we observed that for a considerable number of younger patients, corneal curvature measurements were often directly substituted with either 43 or 43.5 Diopter. Therefore, we chose not to analyze the correlation between corneal curvature and axial length development in this study, deeming it of limited value. Future investigations will include longitudinal follow-ups on the postoperative outcomes of this group of unilateral congenital cataract patients. To build up the findings of this study, future research will focus on longitudinal follow-ups of postoperative outcomes in unilateral congenital cataract patients. Future investigations will include longitudinal follow-ups on the postoperative outcomes of this group of unilateral congenital cataract patients. Future prospective studies with larger sample sizes and more advanced biometry are needed to validate our findings. Additionally, we plan to extend our research to encompass bilateral congenital cataract patients, providing valuable clinical references for the treatment and recovery of children with congenital cataract patients.

## Conclusions

The study highlights the importance of early detection and intervention in congenital cataract to prevent abnormal ocular development. The duration of form deprivation, degree of lens opacity, and history of oxygen exposure are critical factors influencing ALD in children with unilateral congenital cataract. These insights can guide clinical practice in managing and predicting outcomes for affected patients.

## Data Availability

The data supporting the results reported in this article are not publicly available but can be accessed by communicating with the corresponding author.
